# Draft Genome Analysis Offers Insights Into the Mechanism by Which *Streptomyces chartreusis* WZS021 Increases Drought Tolerance in Sugarcane

**DOI:** 10.3389/fmicb.2018.03262

**Published:** 2019-01-09

**Authors:** Zhen Wang, Manoj Kumar Solanki, Zhuo-Xin Yu, Li-Tao Yang, Qian-Li An, Deng-Feng Dong, Yang-Rui Li

**Affiliations:** ^1^Agricultural College, State Key Laboratory of Subtropical Bioresources Conservation and Utilization, Guangxi University, Nanning, China; ^2^Key Laboratory of Sugarcane Biotechnology and Genetic Improvement Guangxi, Ministry of Agriculture, Sugarcane Research Center, Chinese Academy of Agricultural Sciences, Nanning, China; ^3^Sugarcane Research Institute, Guangxi Academy of Agricultural Sciences, Nanning, China; ^4^Department of Postharvest and Food Sciences, Agricultural Research Organization, Volcani Center, Rishon LeZion, Israel; ^5^State Key Laboratory of Rice Biology, Institute of Biotechnology, Zhejiang University, Hangzhou, China

**Keywords:** drought stress, genome, plant growth promotion, *Streptomyces*, sugarcane

## Abstract

Drought directly affects sugarcane production. Plant growth-promoting bacteria have gained attention as growth promoters of plants under abiotic stresses. The present study focused on genome assessment of the plant-beneficial endophyte *Streptomyces chartreusis* WZS021 and its vital role in sugarcane plants under drought stress. Based on *in vitro* plant growth-promoting trait analyses, WZS021 had multiple abilities, including tolerance to drought and production of 1-aminocyclopropane-1-carboxylic deaminase, siderophores, and indole acetic acid. We confirmed root colonization of sugarcane transplants by WZS021 by a sterile sand assay and scanning electron microscopy. Plants inoculated with strain WZS021 had a positive influence on the root parameters such as length and biomass when compared to the control plants. A comparative study of the responses of two sugarcane varieties (ROC22 and B8) to different levels of drought stress in the presence or absence of WZS021 was conducted by assessing the plant chemistry. The expression of antioxidants in sugarcane leaves varied with water stress level. WZS021 inoculation improved the contents of chlorophyll, proline, and phytohormones, revealing some potential for the mechanisms by which this strain improves drought tolerance in sugarcane plants. We identified several genes that might be involved in the plant growth- and drought tolerance-promoting effects of this strain.

## Introduction

Sugarcane is an important sugar and energy crop that is utilized as a raw material in various industries. The sugar industry is the pillar of the economy in Guangxi Province. Guangxi is the largest sugar-producing province in China; it produces >60% of total sugar in the country (Li Y.R. et al., 2016). However, >80% of sugarcane in China is cultivated in dry, sloping fields that lack adequate irrigation systems. Furthermore, drought has become an major factor limiting sugarcane production (Li and Yang, 2015). Bio-fertilizers to promote sugarcane growth under stress conditions are critically needed. Plant growth-promoting rhizobacteria (PGPR), known as beneficial endophytic or root-associated microflora, can improve plant growth by promoting soil nutrient availability and uptake. PGPR have been reported to improve drought tolerance in *Arabidopsis* (Timmusk and Wagner, 1999), pepper (Mayak et al., 2004b), tomato (Mayak et al., 2004b), wheat (Yandigeri et al., 2012), and pea (Belimov et al., 2009). Therefore, the utilization of such bacteria might be an effective approach to stabilizing and increasing crop yield and improving water use efficiency in dryland farming (Marris, 2008; Glick, 2014; Gontiamishra et al., 2014).

Salinity and drought are important ecological factors that affect crop yield via alterations in water relations, ionic homeostasis, and metabolic perturbations, and by inducing the generation of reactive oxygen species (ROS) and causing tissue damage. Under stress conditions, PGPR trigger phytohormone synthesis, inhibit the growth of plant pathogens, and induce systemic resistance (Malik et al., 1997; Sathya et al., 2017). Yang et al. (2009) proposed the term “induced systemic tolerance” (“IST”) to refer to defense-related physical and chemical changes in plants induced by microbes under abiotic stresses. During IST, endogenous phytohormones regulate the metabolic activities of plants, depending on environmental factors. Under stress, the metabolic pathways in plants are either up- or downregulated at various developmental stages, thus affecting plant growth (Chaves et al., 2002). Similarly, under drought stress, endogenous phytohormones regulate plant growth in order to adapt to the environmental changes (Wolters and Jürgens, 2009). The mechanisms of action of most phytohormones in the regulation of plant growth and development are very complex, and a single phytohormone can regulate multiple developmental processes. Furthermore, a single developmental process might require synergistic effects of various phytohormones (Xiong et al., 2009). Certain phytohormones, such as indole-3-acetic acid (IAA), cytokinin (CTK), and gibberellin (GA), can be synthesized by PGPRs as well as plants (Tien et al., 1979; Nieto and Wtjr, 1989). These hormones promote plant growth, enhance root development, increase water and fertilizer absorption, and participate in the regulation of different metabolic activities in plants in response to damage (Graham, 2003; Stepanova et al., 2007).

Filamentous bacteria (Actinobacteria) might help improve the resilience of plants under water (Kohler et al., 2008; Yandigeri et al., 2012), salt (Mayak et al., 2004a; Ryu et al., 2004; Cheng et al., 2007), and heavy metal stresses (Wu et al., 2006; Dell’amico et al., 2008). *Streptomyces* species reportedly can produce 1-aminocyclopropane-1-carboxylate (ACC) deaminase, IAA, and siderophores, which can improve plant growth, under stress conditions (Palaniyandi et al., 2014). For example, *Streptomyces* sp. can promote growth in maize (Aly et al., 2003), wheat (Aly et al., 2012), *Arabidopsis* (Palaniyandi et al., 2014), and chickpea (Srivastava et al., 2015) under saline stress. Similarly, drought-tolerant *Streptomyces* sp. enhanced yield in wheat (Yandigeri et al., 2012). The mechanisms underlying the drought tolerance-promoting effects of *Streptomyces* sp. in plants have been documented and include increasing the osmotic pressure in plant cells, callose accumulation, and lignification of the cell walls (Hasegawa et al., 2007, 2008). The commercial usability of Actinobacteria as a bio-fertilizer has been validated (Hayat et al., 2010). To improve the bio-fertilizer technology, it is essential to understand the molecular mechanisms of plant growth promotion and drought tolerance of Actinobacteria. The identification of genes that contribute to the beneficial activity of Actinobacteria, besides adding to our understanding of the molecular mechanisms, will aid in the development of better bio-fertilizers.

However, the molecular mechanisms underlying drought resistance in Actinobacteria have not been examined in depth. Single molecule real-time (SMRT) analysis and draft-genome annotation can be used to identify genes potentially involved in the beneficial effects of PGPR (Kang et al., 2016; Qin et al., 2017; Oh et al., 2018) and can provide insight into the molecular mechanisms, functional capabilities, and biodiversity of actinobacterial species. Therefore, in the present study, we aimed to (1) compare actinobacterial growth and activity under normal and stressed conditions, (2) evaluate the capacity of Actinobacteria to enhance growth and drought tolerance in sugarcane, and (3) analyze the draft genome sequence of *Streptomyces chartreusis* WZS021, which potentially contributes to plant growth and stress regulation. We expected our draft genome study to add to the current knowledge of the stress tolerance function of actinobacteria in sugarcane.

## Materials and Methods

### *S. chartreusis* Strain and Plant Materials

*Streptomyces chartreusis* strain WZS021 was obtained from the Sugarcane Laboratory of Physiology, Biochemistry and Molecular Biology at Guangxi University (Nanning, China). Sugarcane seedlings of varieties ROC22 and B8 were obtained from the Sugarcane Research Institute of Guangxi Academy of Agricultural Sciences (Nanning, China).

### Determination of *in vitro* Growth Kinetics Under Water Stress

To investigate the bacterial competence under drought stress, we assessed the growth kinetics of strain WZS021 under different water potentials (0, -0.05, and -0.30 MPa) established by mixing the appropriate concentrations of polyethylene glycol (PEG) 6000 (Michel and Kaufmann, 1973) in tryptic soy broth (TSB) (Solarbio Technology Co. Ltd., Beijing, China). In these experiments, WZS021 (∼10^6^ cells mL^-1^) was inoculated in a 500-mL Erlenmeyer flask containing 100 mL of TSB and incubated at 30°C with constant agitation at 250 rpm. We performed the experiment in 21 flasks and 3 flasks were randomly selected in each time point for harvesting the cell biomass. The cell biomass was harvested every day for up to a week. Biomass production was determined by measuring the dry weight after drying the biomass at 105°C.

### Determination of ACC Deaminase Activity

1-ACC deaminase activity in WZS021 was assessed based on its ability to use ACC as a sole nitrogen source, by spot inoculation on Dworkin and Foster (DF) salt agar medium (Honma and Shimomura, 1978), after confirmation of growth on DF medium. Strain WZS021 was grown in 5 mL of TSB at 30°C until the stationary phase was reached. Then, the cells were collected by centrifugation, washed twice with 0.1 M Tris-HCl (pH 7.5), and suspended in 2 mL of modified DF minimal medium supplemented with 3 mM ACC and PEG 6000 to establish different water potentials (0, -0.05, and -0.30 MPa). The cells were then incubated at 30°C with shaking for another 36–72 h. ACC deaminase activity was determined by measuring the production of a-ketobutyrate, which is generated by cleavage of ACC by ACC deaminase (Penrose and Glick, 2003). After determining the amount of protein and a-ketobutyrate, the enzyme activity was expressed as micromoles of a-ketobutyrate mg^-1^ protein h^-1^. Detailed methods are described in the Supplementary Material.

### Determination of PGP Traits

To quantify the production of IAA, pure bacterial culture was inoculated in L-tryptophan broth (1 mL) and grown for 48 h, and then the supernatant was collected by centrifugation (at 8,000 × *g* for 10 min). One milliliter of Salkowski’s reagent and 10 μL of orthophosphoric acid (95%) were added to the supernatant (Gordon and Weber, 1951). The solution was incubated at 37°C for 10 min, and the absorbance at 530 nm was measured using a Multiskan GO microplate reader (Thermo Scientific, Waltham, MA, United States). Siderophores were detected using chrome azurol S (CAS) medium (Alexander and Zuberer, 1991). A CAS plate was inoculated with WZS021 and incubated at 30°C for 5 days. Then, the formation of a yellow halo zone around the colony, indicating siderophore production, was checked. The phosphate-solubilizing ability of the strain was determined by growing it on a plate containing phosphate dissolving medium (Hopebio Biological, Qingdao, China) at 30°C for 5 days. Then, the formation of a halo transparent zone around the colony, indicating phosphate-solubilizing ability, was checked (Oliveira et al., 2009).

### Sand Pot Assay and Electron Microscopy

Strain WZS021 was grown on TSB at 30°C for 5 days. Then, the cells were harvested by centrifugation at 5,000 × *g* for 10 min, washed, and resuspended in sterilized water at ∼10^6^ colony-forming units (cfu) mL^-1^ for inoculation. Sugarcane seedlings were planted in plastic pots (20 cm diameter, 18 cm depth) filled with sterilized fine sand. The pots were moistened with sterilized half strength Hoagland’s nutrient solution and maintained in a greenhouse (26°C/21°C, 16/8-h light/dark cycles). After 3 days, each pot was inoculated with 10 mL of WZS021 suspension directly into the pot, and the control seedlings were watered with sterile water. WZS021 inoculated and control, each treatment consisted of 10 biological replicates. The plants’ roots were collected on days 7, 14, 21, and 28 after inoculation. Root characteristics were examined using a Z2400 root scanner system (Peking University Founder Group, Beijing, China) equipped with WinRHIZO software.

For scanning electron microscopy, root samples collected on days 7 and 14 were used. The samples were washed with phosphate-buffered saline (pH 7.2) three times and fixed with 2.5% glutaraldehyde overnight (Azura et al., 2016), and dehydrated in a graded series of acetone (30, 50, 70, 80, 95, and 100%; 20 min per concentration). Then, the samples were dried using hexamethyldisilazane, coated with gold using ion sputtering equipment (EM ACE200; Leica Microsystems, Solms, Germany), and observed under a scanning electron microscope (SU8020; Hitachi High-Tech Instruments, Saitama, Japan).

### Pot Experiment Under Drought Condition

The pot experiment was carried out in the automatic greenhouse of Agricultural College, Guangxi University. Pots (40 cm diameter, 35 cm depth) were filled with soil (0.151% total N, 0.079% total P, 2.278% total K, 52 mg kg^-1^ available N, 46 mg kg^-1^ available P, 141 mg kg^-1^ available K, 13.36 g kg^-1^ organic matter; pH 7.3 and 25.9% soil moisture). Three water potentials (0, -0.05, and -0.30 MPa) were used, with ten pots per treatment. Sugarcane stem sets with a single bud were soaked in water at 52°C for 30 min and then in bacterial suspension (∼10^6^ cfu mL^-1^) at room temperature for 1 h, and were then planted. Fresh bacterial suspension (200 mL) was inoculated once in 2 months until the elongation stage (up to 5 months) (Li C. et al., 2016). Water stress treatments were initiated after the start of the elongation stage (Table 1). After determining the initial water content in the soil, the soil water content was measured daily by the weighing method and was maintained within the set range.

**Table 1:** Different range of water stress used as treatments in the greenhouse experiment.

Water stress degree	Stress days	Soil water content (%)
Control (CK)	0	80 ± 5
Mild drought (D1)	3	60 ± 5
Moderate drought (D2)	6	45 ± 5
Severe drought (D3)	9	30 ± 5
Re-water 1d (Rw1)	-1	80 ± 5
Re-water 3d (Rw2)	-3	80 ± 5


### Assessment of Antioxidants, Chlorophyll, Proline, and Phytohormones

After 5 months, plant leaves were collected and were ground to a fine powder with liquid nitrogen. To determine the activity of superoxide dismutase (SOD), the optical density at 560 nm of the reaction mixture after photochemical nitroblue tetrazolium reduction was determined (Giannopolitis and Ries, 1977). Peroxidase (POD) activity was determined by the guaiacol method (Sakharov and Ardila, 1999). Catalase (CAT) activity was determined using a detection kit (product no. A007; Nanjing Jiancheng Bioengineering Institute, China), according to the manufacturer’s protocol. Malondialdehyde (MDA) activity was determined by measuring thiobarbituric acid reaction product (Ohkawa et al., 1979). In brief, powdered leaves (3 g) was treated with acetocaustin and boiled in thiobarbituric acid for 10 min. After centrifugation at 5,000 × *g* for 5 min, the absorbance of the supernatant at 532, 600, and 450 nm was measured. Total chlorophyll content was measured directly from a plant leaf using a chlorophyll meter (SPAD-502; Konica Minolta Co. Ltd., Tokyo, Japan). Proline content was estimated according to the method described by Bates et al. (1973). Briefly, 0.1 g of leaf was placed in a large test tube with 5 mL of 3% sulfosalicylic acid that was placed in a water bath shaker at 120 rpm at 100°C for 10 min for extraction. The extract (2 mL) was filtered into a clean test tube, and 2 mL of glacial acetic acid and 2 mL of acetic acid ninhydrin reagent were added. The tube was incubated in boiling water for 30 min, when the solution turned red. After cooling, 4 mL of toluene was added and vortexed for 30 s, and the upper liquid layer was transferred into a 10-mL centrifuge tube and centrifuged at 5,000 × *g* for 5 min. The optical density of the red supernatant at 520 nm was measured, with toluene as a blank. Endogenous phytohormones, including IAA (product no. JL14098), abscisic acid (ABA; product no. JL13378), and ethylene (ET; product no. JL14097) in sugarcane tissues were quantified using enzyme-linked immunosorbent assay (ELISA) kits (Jianglai Biological Science and Technology, Shanghai, China) per the manufacturer’s instructions. All experiments were performed with three biological and technical replicates.

### Genome Sequencing, Assembly, and Annotation

Genomic DNA was extracted from WZS021 cells using the E.Z.N.A.^®^ bacterial DNA Kit (Omega, Norcross, GA, United States). DNA quality and concentration were evaluated using a NanoDrop ND-2000 UV-vis spectrophotometer (Thermo Scientific, Wilmington, DE, United States), based on the ratios 260/280 nm and 260/230 nm. Genome sequencing was conducted by Novogene Bioinformatics Technology (Beijing, China), using the PacBio RS II SMRT sequencing platform. A 10-kb genomic library was constructed. Detailed information on genome assembly is presented in the Supplementary Material. Low-quality reads were filtered out using SMRT software version 2.3.0 (Berlin et al., 2015; Koren and Phillippy, 2015), and the filtered reads were assembled to generate contigs. Gene functions were predicted using seven databases: Gene Ontology (GO) (Ashburner et al., 2009), Kyoto Encyclopedia of Genes and Genomes (KEGG) (Kanehisa et al., 2004; Kanehisa et al., 2006), Clusters of Orthologous Groups (COG) (Tatusov et al., 2006), Non-Redundant Protein Database databases (NR) (Li et al., 2002), Transporter Classification Database (TCDB) (Saier et al., 2014), Swiss-Prot (Bairoch and Apweiler, 2000), and TrEMBL (Michele and Uniprot, 2011). Secondary metabolism-related gene clusters were analyzed using antiSMASH (Medema et al., 2011). The genome sequence data were submitted to GenBank under BioProject PRJNA448493 (accession number QVOF00000000) and all 22 contigs were submitted under accession numbers QVOF01000001–QVOF01000022.

### Statistical Analysis

IBM^®^ SPSS^®^ Statistics V21 software was used to analyze the data obtained. All experiments were performed in triplicate. Data are reported as the mean and the standard error of the mean. Means were compared by the Student *t*-test. Heatmaps were generated based on growth responses of sugarcane roots by the Euclidean distance method using CIMminer software. OriginPro 2018 v9.5.1 was used for graph plotting and three-way multivariate analysis (MANOVA). A *p*-value < 0.05 was considered significant.

## Results

### WZS021 Growth Kinetics and PGP Traits

**FIGURE 1:**
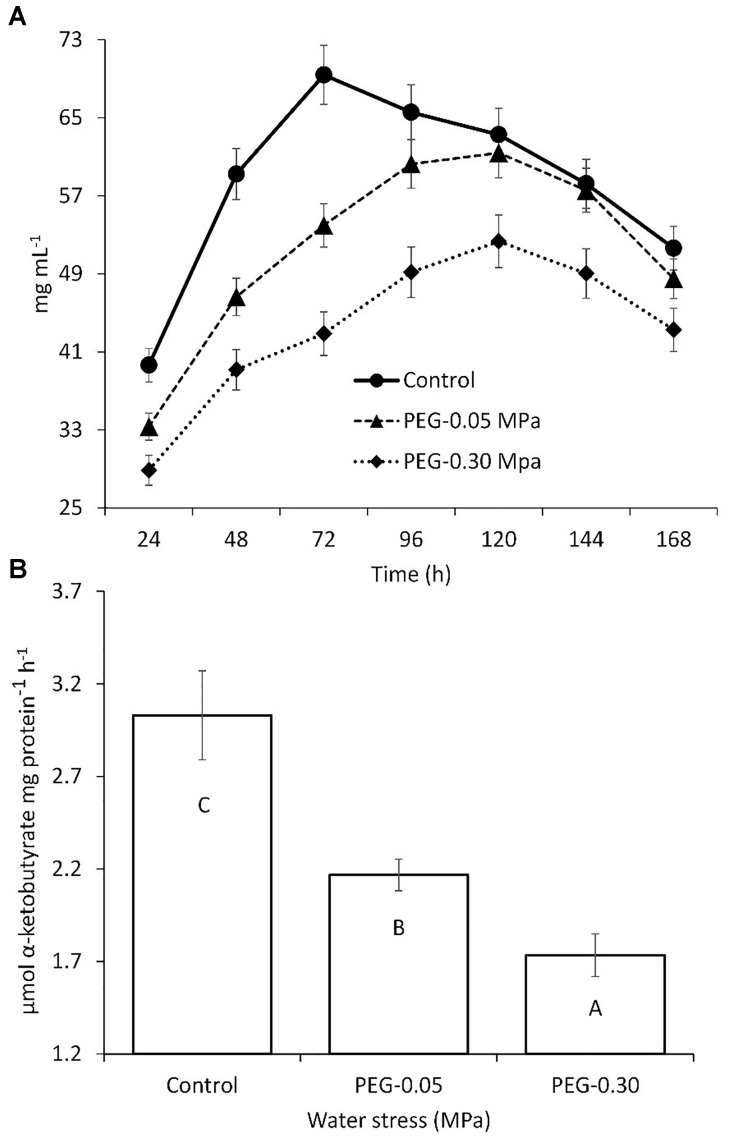
Comparison of growth **(A)** and ACC deaminase production **(B)** of actinobacterial strain WZS01 under different range of water stress. Experiment repeated three times and mean (*n* = 3) with standard error were used and significance (*p* < 0.01) among treatments were calculated by Duncan’s Multiple Range Test.

*Streptomyces chartreusis* strain WZS021 was screened for drought tolerance using PEG 6000. The growth of WZS021 was affected by water stress induced by PEG 6000; however, the cells were able to grow at a water potential of -0.30 MPa (Figure 1A). Cellular dry weight declined with increasing water stress. ACC deaminase activity in strain WZS021 was assessed under normal and severe drought stress conditions (-0.05 and -0.30 MPa, respectively). The ACC deaminase activity was low under drought stress, and decreased by 28–42% (Figure 1B). WZS021 produced IAA (up to 30.5 ± 0.59 mg mL^-1^) and siderophores (up to 5.19 ± 0.43 mm).

### Effect of Strain WZS021 on Root Morphology

**FIGURE 2:**
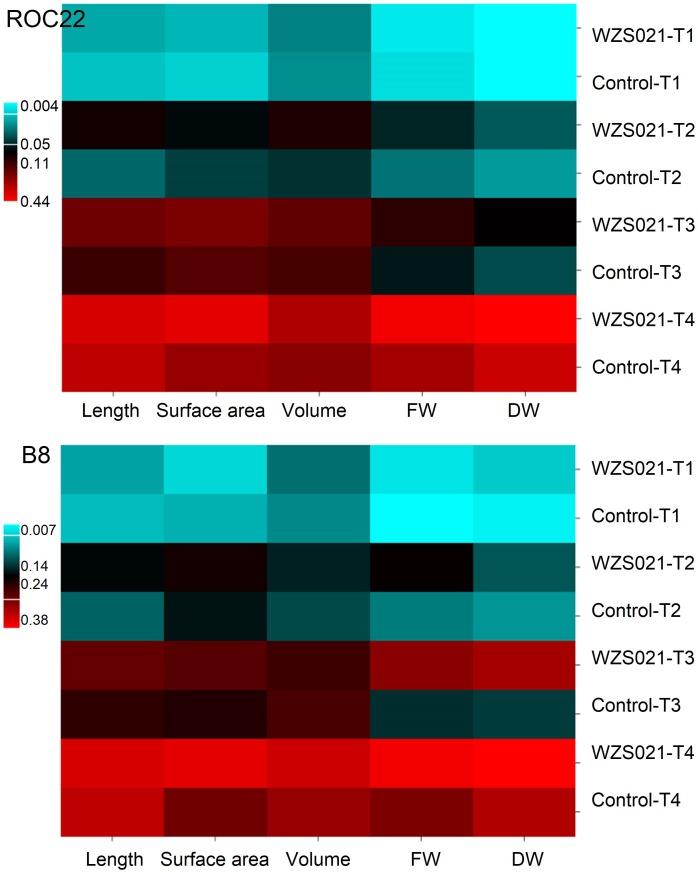
Effect of actinobacterial strain WZS021 on root physical parameters in two sugarcane varieties, ROC22 and B8. Heat map grafted through the growth responses of root parameters. FW, fresh weight; DW, dry weight, T1–T4: 7, 14, 21, and 28 days after inoculation (DAI).

**FIGURE 3:**
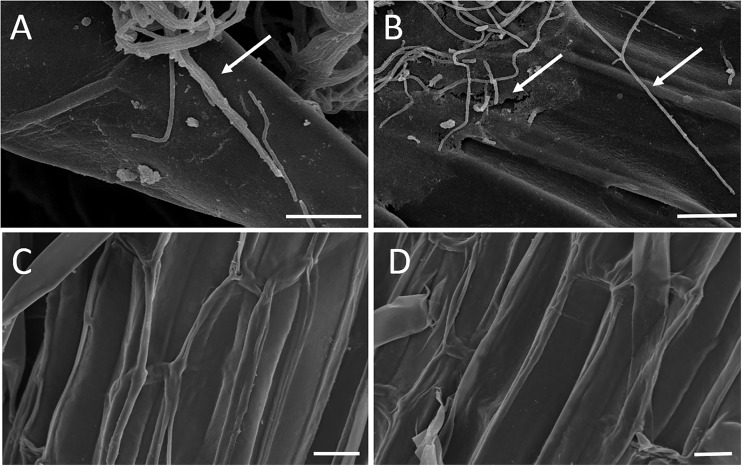
Mycelia of actinobacterial strain WZS021 growing on the root surface of two sugarcane varieties ROC22 **(A)** and B8 **(B)**, control of ROC22 **(C)**, and B8 **(D)**. Bars indicate 10 μm.

The results of the sand pot assay showed that strain WZS021 has a positive effect on root length, surface area, volume, and weight when compared to the control, in both sugarcane varieties tested (Figure 2). The heatmap in Figure 2 reveals that growth responses of WZS021-inoculated and control seedlings were clearly differentiated. In ROC22, WZS021 significantly enhanced root length at T1 (*p* < 0.01), T2 (*p* < 0.01), and T3 (*p* < 0.05), and root weight (fresh and dry) at T2 (*p* < 0.05), T3 (*p* < 0.05), and T4 (*p* < 0.001), and root volume at T4 (*p* < 0.01) (Figure 2 and Supplementary Table S1). In B8, WZS021 significantly (*p* < 0.05–0.001) enhanced root length and weight (fresh and dry) at T1, T2, T3 and T4, and root volume at T1 and T4, and surface area at T2 and T4, respectively (Figure 2 and Supplementary Table S1). Scanning electron microscopy revealed that the WZS021 can colonize the roots of both sugarcane varieties ROC22 and B8 (Figure 3). WZS021 mycelium grew on and was attached to root and root-hair surfaces.

### Greenhouse Experiment

**FIGURE 4:**
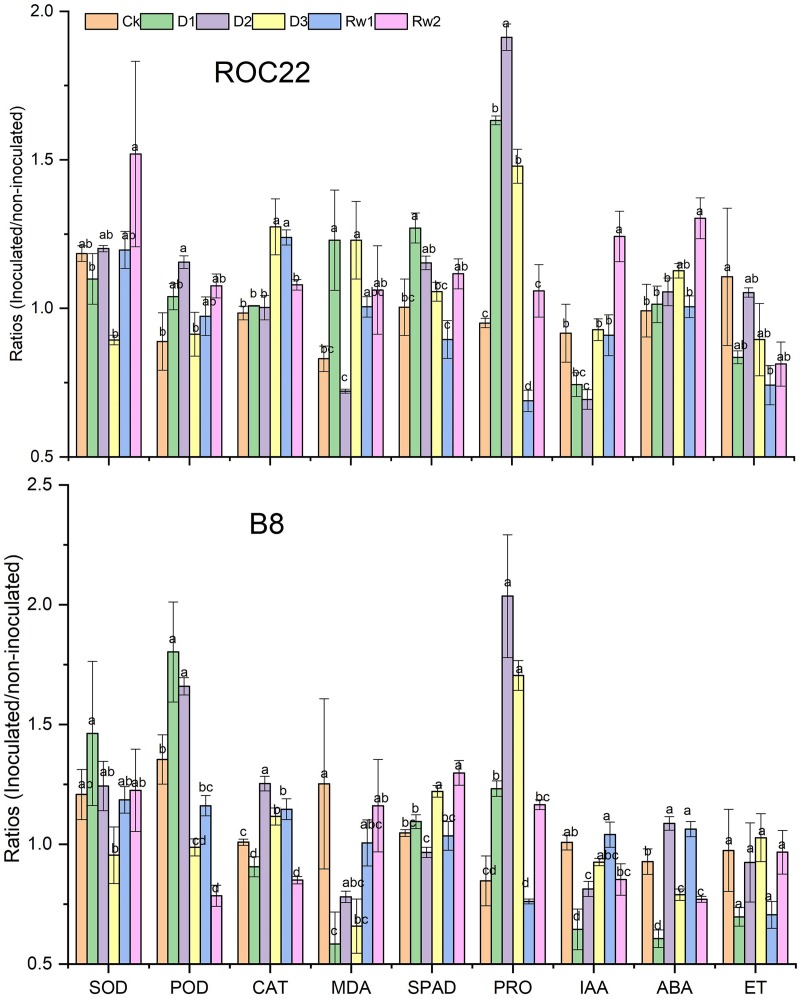
Bars indicate the ratios (inoculated/un-inoculated) of antioxidants, chlorophyll, proline, and phytohormones in two sugarcane varieties ROC22 and B8. Mean (*n* = 3) with standard error was used and different letter shows significance (*p* < 0.01) among treatments based on Least significance different (LSD) test. SOD, superoxide dismutase; POD, peroxidase; CAT, catalase; MDA, malondialdehyde; SPAD, chlorophyll content; IAA, indole acetic acid; ABA, abscisic acid and ET, ethylene.

To understand the beneficial effect of strain WZS021 on the sugarcane varieties ROC22 and B8, seedlings were grown with or without WZS021 under different drought stress conditions (Supplementary Figure S1), and antioxidants, chlorophyll, proline, and phytohormones ratios (inoculated/uninoculated) were compared (Figure 4). Supplementary Figure S1 shows the different kind of impact of bacterial inoculation on plant growth under different drought stress conditions.

#### Impact of WZS201 Inoculation in Antioxidant Production

Drought stress enhances the deleterious effects of ROS in plant cells. To avoid harmful intracellular ROS concentrations, plants activate antioxidative defense systems operated by antioxidants, such as SOD, POD, CAT, and MDA (Figure 4). Under severe drought stress (D3), SOD activity decreased significantly (*p* < 0.05) by 25% in ROC22 and by 21% in B8 as compared to the levels in well-watered control plants (Figure 4). SOD activity increased under mild stress (D1) in B8 (by 21%) and at Rw2 in ROC22 (by 28%) as compared to the levels in well-watered control plants (Figure 4). POD and CAT activities increased under drought stress. POD activity increased by up to 30% in ROC22 and by 21% in Rw2 (21%) under moderate stress (D2), and by 17% in ROC22 under mild drought stress (D1). CAT activity increased by 30% in ROC22 during D3 and by 26% at Rw1 when compared to the levels in well-watered control plants. Similarly, in B8, POD activity increased during D1 (by 33%) and D2 (by 23%), whereas CAT activity increased during D2 (by 24%), at Rw1 (by 14%), and D3 (by 11%). POD activity decreased significantly during D3 (by 27%) and at Rw2 (by 14%), whereas CAT activity decreased during Rw2 (by 16%) and D1 (by 10%). Drought stress treatment significantly influenced MDA in both varieties. In ROC22, MDA decreased under D2 (by 13%) and increased significantly under D1 and D3, whereas in B8, MDA was significantly lower under treatments D1 and D3 than in the well-watered control (Figure 4).

#### Impact of WZS201 Inoculation in Chlorophyll Production

Chlorophyll is a well-known growth indicator of plant health. Drought stress significantly affected the chlorophyll content of both varieties in the presence of strain WZS021. The chlorophyll content (SPAD value) of ROC22 increased under D1 (by 27%), D2 (by 15%), and Rw2 (11%) when compared to the contents in well-watered control plants, whereas B8 showed significant increases in chlorophyll content at Rw2 (24%) and under D3 (17%), and a significant decrease under D2, when compared to the control (Figure 4).

#### Impact of WZS201 Inoculation in Proline Production

To manage osmotic stress enforced by drought, plants synthesize osmolytes, such as proline, in the cytosol. Drought stress treatment significantly enhanced the proline contents in both varieties in each of D1, D2, and D3. At Rw1, proline content was low in both varieties when compared to the levels in control plants (Figure 4).

#### Impact of WZS201 Inoculation in Phytohormones Production

As IAA, ABA, and ET modulate plant growth in drought stress, we quantified these phytohormones. Drought stress significantly affected IAA accumulation in ROC22 and B8 seedlings; ROC22 seedlings had low IAA content under treatments D1 (19%) and D2 (24%), and significantly increased content at Rw2 when compared to control plants (Figure 4). Likewise, B8 seedlings showed significantly low IAA content under treatments D1 (36%) and D2 (19%). ABA accumulation was also strongly affected by drought stress. ROC22 seedlings had higher ABA contents than control plants, whereas B8 had lower ABA contents during D1, D3, and at Rw2. B8 seedlings had higher ABA contents than control plants under D2 and Rw1 (Figure 4). No significant effect of strain WZS021 on ET was observed in B8, whereas ROC22 showed a decrease in ET as compared to the control, and the decrease was significant under treatment Rw1 (Figure 4).

### Three-Way ANOVA of Antioxidants, Chlorophyll, Proline, and Phytohormones

To determine the interaction effects of variables (two cultivars, with or without WZS021, and drought stresses), we conducted three-way MANOVA. Factorial ANOVA showed that WZS021 inoculation and drought stress had a significant effect on the plant parameters measured (Table 2). Moreover, except for SOD and MDA, cultivar also had a significant effect on the plant parameters. The interaction between cultivar and WZS021 inoculation had a significant effect only on ABA, POD, and MDA. Conversely, cultivar and drought stress, and drought stress and WZS021 inoculation, showed significant interaction effects on all plant parameters measured. Additionally, there were significant interaction effects of cultivar, WZS021 inoculation, and drought stress on POD, ABA, proline, CAT, MDA, IAA, and SPAD (Table 2).

**Table 2:** Summary of three-way ANOVA for the effects of two cultivar, with or without WZS021 and different gradient of drought stresses on antioxidants, chlorophyll, proline, and phytohormones of sugarcane plant.

Variables		SOD	POD	CAT	MDA	SPAD	Proline	ET	ABA	IAA
Cultivar (DF-1)	F	2.34	265.71	56.23	0.02	21.59	108.91	35.77	38.08	63.86
	Sig.	0.13^ns^	^∗∗∗^	^∗∗∗^	0.88^ns^	^∗∗∗^	^∗∗∗^	^∗∗∗^	^∗∗∗^	^∗∗∗^
WZS021(DF-1)	F	21.53	91.65	24.86	9.00	24.78	207.48	17.31	17.69	55.53
	Sig.	^∗∗∗^	^∗∗∗^	^∗∗∗^	^∗∗^	^∗∗∗^	^∗∗∗^	^∗∗∗^	^∗∗∗^	^∗∗∗^
Drought (DF-5)	F	20.84	258.17	36.48	11.95	78.48	213.92	26.24	72.04	44.76
	Sig.	^∗∗∗^	^∗∗∗^	^∗∗∗^	^∗∗∗^	^∗∗∗^	^∗∗∗^	^∗∗∗^	^∗∗∗^	^∗∗∗^
Cultivar × WZS021 (DF-1)	F	0.19	81.95	2.00	4.05	0.41	0.69	0.42	93.49	2.08
	Sig.	0.67^ns^	^∗∗∗^	0.16^ns^	^∗^	0.53^ns^	0.41^ns^	0.52^ns^	^∗∗∗^	0.16^ns^
Cultivar × Drought (DF-5)	F	6.90	56.18	4.93	10.99	2.94	124.89	3.43	25.26	5.68
	Sig.	^∗∗∗^	^∗∗∗^	^∗∗^	^∗∗∗^	^∗^	^∗∗∗^	^∗∗^	^∗∗∗^	^∗∗∗^
WZS021 × Drought (DF-5)	F	2.74	44.91	11.76	2.82	4.31	91.76	3.14	17.16	15.72
	Sig.	^∗^	^∗∗∗^	^∗∗∗^	^∗^	^∗∗^	^∗∗∗^	^∗^	^∗∗∗^	^∗∗∗^
Cultivar × WZS021 × Drought (DF-5)	F	0.56	27.92	8.42	6.21	3.19	9.86	1.13	23.26	5.31
	Sig.	0.73^ns^	^∗∗∗^	^∗∗∗^	^∗∗∗^	^∗^	^∗∗∗^	0.36^ns^	^∗∗∗^	^∗∗∗^


*DF, degrees of freedom. Sig., significance level (ns, not significant, ^∗^*p* < 0.05, ^∗∗^*p* < 0.01, ^∗∗∗^*p* < 0.001), SOD, superoxide dismutase; POD, peroxidase; CAT, catalase; MDA, malondialdehyde; SPAD, chlorophyll content; IAA, indole acetic acid; ABA, abscisic acid and ET, ethylene.*

### Genome Properties and Genes Related to Growth and Water Stress

**FIGURE 5:**
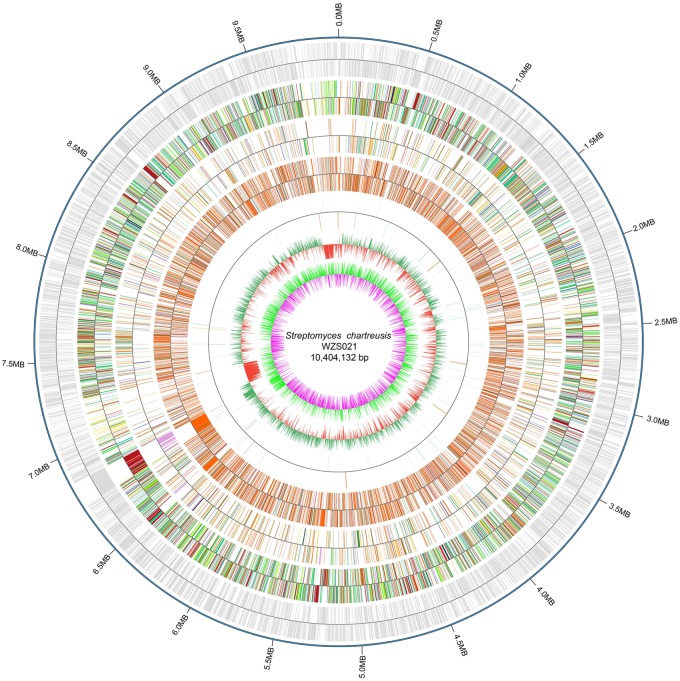
The overview of genome map of actinobacterial strain WZS021. From outside to center: scale marks, CDSs, COG, KEGG, GO, ncRNA, GC content and GC skew.

**FIGURE 6:**
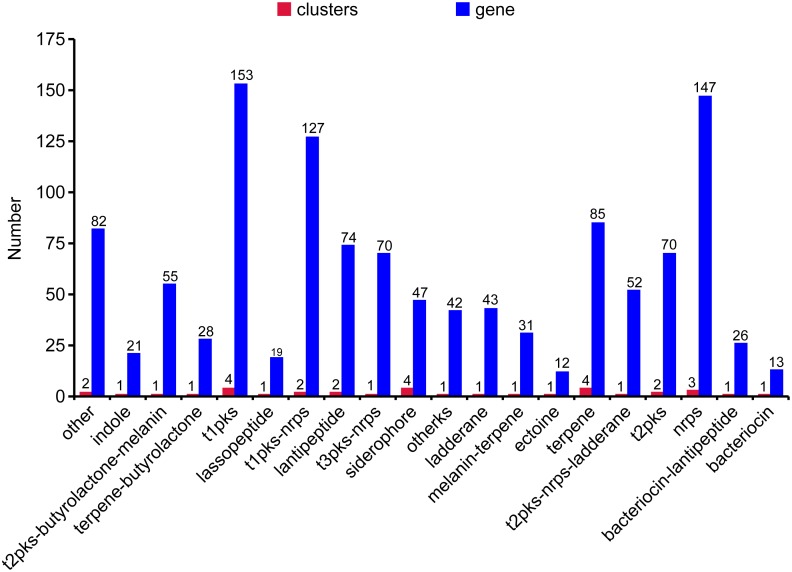
The secondary metabolites gene clusters of actinobacterial stain WZS021 genome.

The genome of WZS021 was sequenced using SMRT technology, generating 99,198 reads. The mean read length was 12,028 bp and the average coverage depth was 80×. General characteristics of the genome are listed in Table 3. Strain WZS021 contains 9,561 predicted protein-coding genes, 17 sRNA genes, 21 rRNA genes, and 72 tRNA genes, which were associated with COG, KEGG, and GO functional categories (Figure 5 and Supplementary Figures S2–S4). The overall G+C content is 71.34%. Analysis of the putative CDSs revealed the genome contains genes that encode proteins involved in nitrogen fixation, ACC deaminase, and IAA secretion (Table 4). The genes *pstA* and *pstC* are required for phosphate transmembrane transporters, and two other important genes in phosphate transport, *pstS* and *pstB*, are responsible for substrate binding and energy supply, respectively. Hydrolase genes, including cellulase, chitinase, xylanase, glucoamylase, α-amylase, malto-oligosyltrehalose trehalohydrolase, and lipase, were also identified. The genome has oxidoreductase genes encoding SOD, glutamate dehydrogenase, succinate-semialdehyde dehydrogenase, proline dehydrogenase, and choline dehydrogenase, which contribute to plant resistance to stress. Further, genes encoding Na^+^, Ca^2+^, and K^+^ transporters, which are involved in plant growth promotion, were detected (Table 4). There were 35 secondary metabolism-related gene clusters in the genome (Figure 6).

**Table 3:** Genome properties of actinobacterial strain WZS021.

Attribute	Value
Number of contigs	22
Genome size (bp)	10,404,132
GC content (%)	71.34
sRNA	17
rRNA	21
tRNA	72
Protein-coding genes (CDS)	9,561
Secondary metabolite clusters	35
Genomic islands	27
Genes assigned to COGs	6,497
Genes assigned to GOs	6,097
Genes assigned to KEGGs	3,971
Proteins with signal peptides	566
Proteins with transmembrane helices	2,076
CRISPR	5
Average coverage (×)	80


**Table 4:** Genes potentially associated with PGP and drought tolerance in actinobacterial strain WZS021 genome.

Gene name	EC NO.	Annotation	^a^Location
*nifU*	-	Nitrogen fixation protein NifU	Contig6: 1111049-516, +
*acdS*	3.5.99.7	1-aminocyclopropane-1-carboxylate deaminase	Contig6: 1137101-8117, +
*iaaM*	1.13.12.3	Tryptophan 2-monooxygenase	Contig14: 436093-7796, +
*trkA1*	-	Trk system potassium uptake protein TrkA	Contig7: 150076-753, -
*trkA2*	-	Trk system potassium uptake protein TrkA	Contig7: 150753-1343, -
*gdh*	1.4.1.2	Glutamate dehydrogenase	Contig3: 456561-61492, -
*pit*	-	Inorganic phosphate transporter, PiT family	Contig3: 1149688-50686, +
*pstA*	-	Phosphate transport system permease protein	Contig3: 1144956-6017, +
*pstC*	-	Phosphate transport system permease protein	Contig3: 1144099-959, +
*pstS*	-	Phosphate transport system substrate-binding protein	Contig3: 1142724-3857, +
*pstB*	-	Phosphate transport system ATP-binding protein	Contig3: 1146072-848, +
*sod*	1.15.1.1	Superoxide dismutase	Contig1: 576667-7062, +
*sod*	1.15.1.1	Superoxide dismutase, Fe-Mn family	Contig6: 36444-7085, -
*entS*	-	MFS transporter, ENTS family, enterobactin (siderophore) exporter	Contig16: 383107-4405, -
*celA*	3.2.1.4	Endo-1,4-β-glucanase	Contig3: 251986-2939, -
*celK1*	3.2.1.91	1,4-β-glucancellobiosidase	Contig5: 375-1367, -
*celK2*	3.2.1.91	1,4-β-glucancellobiosidase	Contig5: 1259-2617, -
*bglB*	3.2.1.21	β-glucosidase	Contig3: 209992-11410, +
*bglX*	3.2.1.21	β-glucosidase	Contig6: 432656-4236, +
*bglF*	3.2.1.21	β-glucosides-specific IIA component	Contig14: 527479-928, -
*malZ*	3.2.1.20	α-glucosidase	Contig6: 1086477-8102, +
*aglE*	-	α-glucoside transport system substrate-binding protein	Contig6: 1088150-9481, +
*aglF*	-	α-glucoside transport system permease protein	Contig6: 1089488-90852, +
*aglG*	-	α-glucoside transport system permease protein	Contig6: 1090849-1688, +
*xynA*	3.2.1.8	Endo-1,4-β-xylanase	Contig16: 271948-3330, -
*glaA*	3.2.1.3	Glucoamylase	Contig14: 19545-21347, -
*amyA*	3.2.1.1	α-amylase	Contig6: 699205-700584, +
*chiC*	3.2.1.14	Chitinase	Contig14: 463794-5032, -
*phzE*	-	Phenazine biosynthesis protein phzE	Contig6: 829073-30962, -
*gabD*	1.2.1.16	Succinate-semialdehyde dehydrogenase (NADP^+^)	Contig1: 1173817-5430, -
*gabT*	-	4-aminobutyrate aminotransferase/(S)-3-amino-2-methylpropionate transaminase	Contig1: 114960-6294, +
*nhaA*	-	Na^+^/H^+^ antiporter, NhaA family	Contig1: 1632278-3717, +
*mnhB*	-	Multicomponent Na^+^/H^+^ antiporter subunit B	Contig3: 1125318-596, +
*mnhC*	-	Multicomponent Na^+^/H^+^ antiporter subunit C	Contig3: 1124049-909, -
*mnhD*	-	Multicomponent Na^+^/H^+^ antiporter subunit D	Contig3: 1123831-4052, -
*mnhF*	-	Multicomponent Na^+^/H^+^ antiporter subunit F	Contig3: 1127872-8648, +
*chaA*	-	Ca^2+^/H^+^ antiporter	Contig6: 1048548-9648, +
*kdpA*	-	K^+^-transporting ATPase ATPase A chain	Contig18: 734321-5982, -
*kdpB*	-	K^+^-transporting ATPase ATPase B chain	Contig18: 732189-4321, -
*kdpC*	-	K^+^-transporting ATPase ATPase C chain	Contig18: 731514-2182, -
*kdpD*	-	Two-component system, OmpR family, sensor histidine kinase KdpD	Contig18: 738354-40819, -
*kdpE*	-	Two-component system, OmpR family, KDP operon response regulator KdpE	Contig7: 148087-770, +
*opuD*	-	Glycine betaine transporter	Contig7: 1290843-2471, +
*treZ*	3.2.1.141	Maltooligosyltrehalose trehalohydrolase	Contig7: 519440-21185, -
*proDH*	1.5.99.8	Proline dehydrogenase	Contig1: 271970-2854, -
*proV*	-	Glycine betaine/proline transport system ATP-binding protein	Contig1: 1079765-80868, -
*proW*	-	Glycine betaine/proline transport system permease protein	Contig1: 1077816-9765, -
*proX*	-	Glycine betaine/proline transport system substrate-binding protein	Contig1: 1076855-7814, -
*plcC*	-	Phospholipase C	Contig7: 1215975-8026, +
*xdhA*	-	Xanthine dehydrogenase small subunit	Contig7: 648675-9565, +
*xdhC*	-	Xanthine dehydrogenase accessory factor	Contig7: 654207-5364, +
*esl*	3.1.1.-	Esterase/Lipase	Contig3: 1159510-60544, -
*betA*	1.1.99.1	Choline dehydrogenase	Contig18: 66766-8439, -
*betB*	-	Betaine-aldehyde dehydrogenase	Contig1: 135743-7182, -
*cspA*	-	Cold shock protein (β-ribbon, CspA family)	Contig3: 1004554-760, -


## Discussion

Drought is the most damaging abiotic stress limiting crop yield worldwide (Dai, 2011). Sugarcane is a major crop for sugar and ethanol production. It has a relatively high water requirement for growth and it is highly sensitive to drought (Li C. et al., 2016). However, microbes promoting stress tolerance in plants are gaining interest in agriculture as a promising alternative to chemicals (Nadeem et al., 2016; Qin et al., 2017). Several studies have already been conducted on PGP microbes, which have vital PGP traits, such as IAA production, siderophore production, nitrogen fixation, nutrient immobilization, iron chelation, and ACC deaminase activity. Furthermore, the systemic resistance induced by microbes can help improve plant health in extreme environments or in the presence of pathogens (Yandigeri et al., 2012; Sathya et al., 2017). Our previous study revealed that WZS021 can fix nitrogen and promote sugarcane growth (Wang et al., 2017). *In vitro* drought screening in the current study revealed that WZS021 can grow at a water potential of -0.30 MPa, which is in agreement with the findings of an earlier study on *Streptomycetes* sp. (Yandigeri et al., 2012). The drought-tolerant PGPR *Gluconacetobacter diazotrophicus* has been reported to promote the growth of sugarcane under water stress (Vargas et al., 2014). In the present study, WZS021 strongly induced ACC deaminase activity, even under water stress. Penrose and Glick (2001) reported that a PGPR containing ACC deaminase reduced the content of ethylene (ET) in plants and promoted root elongation. Bergner and Teichmann (1993) reported that under drought stress, ET production increased, which caused a significant reduction in plant growth. Moreover, apart from ACC deaminase, strain WZS021 has other PGP traits, including siderophore production (Solanki et al., 2014), IAA production (Solanki et al., 2016), and antagonistic activity against sugarcane pathogens, rendering this strain potentially very useful.

To prove that WZS021 can colonize plant roots and promote plant growth, we performed an *in vivo* root colonization assay. Scanning electron microscopy revealed that the strain has significant root colonization ability. These results are in agreement with the findings of earlier studies on PGPRs. Gopalakrishnan et al. (2014) reported that root-colonizing Actinobacteria have the ability to promote plant growth. Furthermore, Yandigeri et al. (2012) found that *Streptomyces olivaceus* DE10 colonized wheat root and promoted plant growth. Accordingly, in the present study, WZS021 inoculation of seedlings had a positive impact on root parameters, as indicated by the sand pot assay, suggesting that WZS021 has significant PGP activity under well-watered condition.

Furthermore, the *in vivo* pot experiment under different levels of water stress indicated that the strain improved plant stress tolerance by regulating the contents of antioxidants. Drought stress induces cellular production of ROS, which can oxidize multiple cellular components, such as proteins, lipids, DNA, and RNA, ultimately causing cell death (Barrera, 2012). In response, plants activate various physiological and biochemical reactions involving antioxidants and phytohormones (Duan et al., 2007). POD, SOD, and CAT are important protective enzymes in plants, and they play an important role in plant defense (Scandalios, 1993). In the present study, WZS021 significantly enhanced the activities of POD, SOD, and CAT in sugarcane plants under moderate drought stress conditions, indicating that this strain can improve plant defense against drought stress. Both sugarcane varieties B8 and ROC22 showed relatively low MDA content under moderate drought when inoculated with WZS021, whereas the content of MDA in B8 decreased throughout the stress period. MDA is a product of membrane lipid peroxidation, and changes in its content reflect the degree of injury to plant cells (Akita and Cabuslay, 1990). Therefore, inoculation of WZS021 more effectively protects the cell membranes from injury in B8, compared to ROC22, by reducing the MDA content. Three-way ANOVA revealed significant interactive effects of WZS021 inoculation with POD, CAT, and MDA on both cultivars under different water-stress levels, indicating that strain WZS021 modulates plant metabolic activities to tolerate drought.

Proline accumulation is a primary response to drought stress in several plant species (Hong et al., 2000). It lowers the water potential of plant tissues, especially in the leaves, enabling them to prevent water loss under drought conditions. The substantially high proline levels observed in WZS021-inoculated plants might help improve plant metabolism under drought stress.

Water stress negatively affected the chlorophyll content in control plants, whereas Actinobacteria-inoculated plants had relatively high chlorophyll contents. Significant differences in chlorophyll content were noted in plants under different water stress conditions. Silva et al. (2007) reported that drought stress decreases the leaf chlorophyll content in sugarcane, depending on the variety.

WZS021 significantly affected the contents of phytohormones in both sugarcane varieties. ET production was reduced upon inoculation with WZS021 in both varieties. We speculate that the reduction in ACC deaminase activity might have led to lower levels of precursor ET, and this would be beneficial for the plant to regulate metabolism under drought conditions. Similar results have been reported by Zhang et al. (2018), who found that ACC deaminase-producing bacteria improved drought tolerance in wheat by reducing ET production. Similarly, Ma et al. (2002) reported that ACC deaminase-producing bacteria decreased ET production in rape plantlets. In the present study, under the same treatment and drought stress conditions, the sugarcane varieties ROC22 and B8 showed differences in ABA contents compared with those in control plants, which might be explained by different genotypic structures. ABA affects plant cell division and elongation, apical dominance, and the transport and accumulation of photoassimilates (Acharya and Assmann, 2009). Further, ABA plays an important role in the biosynthesis system in plants by regulating the growth and development of plants under stress (Borel et al., 2001; Sauter et al., 2001; Jia et al., 2002; Sharp and Lenoble, 2002). The differential patterns of ABA accumulation in the current study did not explain its role in defense against drought stress. Therefore, in-depth studies are needed to understand genotypic and bacterial-induced variability. Further, the control plants had relatively high levels of IAA under all stress treatments, indicating that under drought, plants require more IAA in their aboveground system for root elongation and balancing the plant moisture content. The response of IAA to drought stress differs among plant species (Zhang et al., 2014). The balance between IAA and other growth-related substances is disrupted when plants encounter external stress (Pustovoitova et al., 2004). There was a significant interactive effect of WZS021 inoculation with ABA, MDA, and IAA in both cultivars under different drought conditions. However, there was no significant interaction with ET correlated with the reduction or decomposition of ET, which helps the plant to resist drought.

The *in vitro* and *in vivo* characterization revealed that the strain WZS021 is a plant-beneficial actinobacterial strain and that it can help plants to resist drought stress. These results warrant analysis of the genomic map in the future, to obtain an in-depth understanding of the growth-promotion and stress-regulation mechanisms. Several previous studies have reported the importance of genome information to understand the nature of bacterial strains (Van Sluys et al., 2002; Weidner et al., 2003; Pühler et al., 2004). The genome data generated in the current study showed that genes involved in stress regulation and plant growth promotion are present in WZS021. We previously reported that strain WZS021 is able to fix nitrogen and, accordingly, we identified a single nitrogen fixation-related gene, *nifU*, in the current study. nifU protein plays an important role in Fe-S cluster assembly, which is required for nitrogen fixation (Smith et al., 2005). Non-nitrogen-fixing bacteria and eukaryotic organisms also utilize Fe-S cluster biosynthesis to fix nitrogen (Ali et al., 2004). Phospholipase C is a class of important hydrolytic enzymes that is mainly involved in the growth and differentiation of plant cells, hormone signal transduction, responses to biological and abiotic stresses, and the regulation of polar growth processes (Meijer and Munnik, 2003; Abdelhaliem et al., 2016). Xanthine dehydrogenase is involved in the homeostasis of phytohormones, such as CTK, ABA, and IAA, which regulate plant traits and stress resistance (Leydecker et al., 1995; Taylor and Cowan, 2001; Smith and Atkins, 2002; Watanabe et al., 2014). In the present study, the hormone levels in sugarcane plants inoculated with WZS021 were different from in non-inoculated control plants, with a direct or indirect relationship with the expression of corresponding genes. Studies on *Arabidopsis* have shown that exogenous proline and rehydration can induce the expression of *proDH* (Kiyosue et al., 1996; Nakashima et al., 1998), and *proDH* expression is up- or downregulated depending on the stress condition (Dietmar et al., 2010). In the present study, sugarcane seedlings showed higher proline content under drought stress, which may be due to the expression of *proDH* by the PGP strain under stress. In various extreme environments, trehalose plays an important role in stabilizing bioactive substances, such as membranes and proteins, and when the extreme factors are removed, it can help the organism to resurrect (Richards et al., 2002). Transfer of the *cspB* gene from *Bacillus subtilis* to maize significantly improved drought resistance in maize (Castiglioni et al., 2008). Lilius et al. (1996) transferred the *betA* gene from *Escherichia coli* to tobacco (*Nicotiana tabacum* ‘SR1’), and this significantly improved plant salt tolerance. α-Glucosidase is a key enzyme that breaks down carbohydrates (Hemalatha et al., 2016). The degradation of lignocellulose requires a synergy of endo-β-1,4-glucanase (EC 3.2.1.4), exo-β-1,4-glucanase (EC 3.2.1.91), and β-glucosidase (EC 3.2.1.21), the latter of which can hydrolyze cellobiose and cellodextrin to glucose (Sun and Cheng, 2002). The plasma membrane Na^+^/H^+^ antiporter extrudes excess intracellular Na^+^ using the transmembrane proton gradient as a driving force. It is the only plasma membrane protein that mediates Na^+^ efflux, which plays a significant role in plant defense against abiotic stresses, especially under salt stress (Zhu, 2001, 2003). The presence of the above genes in WZS021 suggests that the strain has the potential to help plants grow under different kinds of abiotic stresses, such as drought and salinity. As the information obtained from the partial genome analysis in this study is limited, further proteomic, transcriptomic, and metabolic analyses will have to be conducted in the future to explore the mechanism of plant-microbe symbiosis.

## Conclusion

*Streptomyces chartreusis* strain WZS021 can improve the growth of sugarcane under drought stress by maintaining plant health through different mechanisms (e.g., producing antioxidants and plant hormones, and promoting plant root elongation). The bacterial genome information provided some clues to explain the experimental data; however, further studies, which may be guided by the genome information presented here, are needed to unravel the complex bacteria–plant interaction networks and to elucidate the molecular mechanisms underlying the PGP traits. Field studies are needed to elucidate the usability of the strain in the field before it can be developed as a plant growth regulator for field application as an alternative to chemical fertilizers.

## Author Contributions

ZW, MS, Y-RL, D-FD, and L-TY contributed to the conception of the study and the experimental design. ZW, Z-XY, and Q-LA conducted the experiments and contributed to the sampling. ZW and MS analyzed and interpreted the data and wrote the manuscript. Y-RL, D-FD, and L-TY contributed to the manuscript editing. All authors read and approved the final manuscript.

## Conflict of Interest Statement

The authors declare that the research was conducted in the absence of any commercial or financial relationships that could be construed as a potential conflict of interest.
