# Supplementary material for: Draft Genome Analysis Offers Insights Into the Mechanism by Which Streptomyces chartreusis WZS021 Increases Drought Tolerance in Sugarcane
Source: Front Microbiol. 2019 Jan 9;9:3262. doi: 10.3389/fmicb.2018.03262 (PMC6338045; doi:10.3389/fmicb.2018.03262)
Supplement: Supplementary file 1 [file Table_1.DOCX]

Draft Genome Analysis Offers Insights Into the Mechanism by Which *Streptomyces Chartreusis* WZS021 Increases Drought Tolerance in Sugarcane

Running title: *Streptomyces chartreusis* WZS021 improves sugarcane drought resistance

Zhen Wang^1^, Manoj Kumar Solanki^2, 4^, Zhuo-Xin Yu^1^, Li-Tao Yang^1^, Qian-Li An^3^, Deng-Feng Dong^1*^, Yang-Rui Li^1, 2*^

^1^ Agricultural College, State Key Laboratory of Subtropical Bioresources Conservation and Utilization, Guangxi University, Nanning 540000, China

^2^ Key Laboratory of Sugarcane Biotechnology and Genetic Improvement Guangxi, Ministry of Agriculture, Sugarcane Research Center, Chinese Academy of Agricultural Sciences, Sugarcane Research Institute, Guangxi Academy of Agricultural Sciences, Nanning 540000, China

^3^ State Key Laboratory of Rice Biology, Institute of Biotechnology, Zhejiang University, Hangzhou 310058, China

^4^ Agricultural Research Organization, Department of Postharvest and Food Sciences, The Volcani Center, 68 HaMaccabim Road, Rishon LeZion 7505101, Israel

*** Correspondence**

**Yang-Rui Li**

[liyr5745@126.com](mailto:liyr5745@126.com)

**Deng-Feng Dong**

dongdfxy@163.com

**Material methods (Suplimentary)**

***Detection of α-ketobutyrate (Section ACC deaminase activity 2.3)***

The ACC deaminase activity was determined by measuring the production of α-ketobutyrate and ammonia generated by the cleavage of ACC (Penrose and Glick 2003). The induced actinobacterial cells were harvested by centrifugation at 3,000 *g* for 5 min, washed twice with 0.1 M Tris-HCl (pH 7.5), and re-suspended in 200 μl of 0.1 M Tris-HCl (pH 8.5). The cells were labilized by adding 5% toluene (v/v) and then vortexed at the highest speed for 30 s. Fifty μL of labilized cell suspension was incubated with 5 μL of 0.3M ACC in an eppendorf tube at 30°C for 30 min. The negative control for this assay included 50 μL of labilized cell suspension without ACC, while the blank included 50 μL of 0.1 M Tris- HCl (pH 8.5) with 5 μL of 0.3 M ACC. The samples were then mixed thoroughly with 500 μL of 0.56 N HCl by vortexing, and the cell debris was removed by centrifugation at 12, 000 *g* for 5 min. A 500 μL aliquot of the supernatant was transferred to a glass test tube and mixed with 400 μL of 0.56N HCl and 150 μL of DNF solution (0.1 g 2,4-dinitrophenylhydrazine in 100 mL of 2N HCl), and the mixture was incubated at 28^o^C for 30 min. One mL of 2N NaOH was added to the sample before the absorbance at 540 nm was measured. The concentration of α-ketobutyrate in each sample was determined by comparison with a standard curve generated as follows: 500 μL α-ketobutyrate solutions of 0, 0.01, 0.05, 0.1, 0.2, 0.5, 0.75 and 1 mM were mixed respectively with 400 μL of 0.56 N HCl and 150 μL DNF solution. One mL of 2N NaOH was added and the absorbance at 540 nm was determined as described above. The values for absorbance versus α-ketobutyrate concentration (mM) were used to construct a standard curve. The protein concentration of toluenized cells was determined by the method of Bradford (1976). A 26.5 μL aliquot of the toluene-labilized actinobacterial cell sample used for the ACC deaminase enzyme assay was diluted with 173.5 μL of 0.1 M Tris-HCl (pH 8.0), and boiled with 200 μL of 0.1 N NaOH for 10 min. After the cell sample was cooled to room temperature, the protein concentration was determined by measuring the absorbance at 595 nm immediately after mixing the solution with 200 μL of Bradford’s reagent. Bovine serum albumin (BSA) was used to establish a standard curve.

***Genome sequencing, assembly, and annotation (Section 2.8)***

After sequencing, quality reads were obtained by removing low-quality bases (mass value ≤ 38) over a certain ratio (default is 40 bp) and also removed the N bases to reach a certain proportion of reads (default is set to 10 bp). Then, removal of overlapped reads performed (default is 15bp), and all qualified reads assembled by the SMRT portal (Version 2.3.0) software (Konstantin et al., 2015; Sergey and Adam, 2015) thought three steps, Initial assembly of the reads using SMRT portal software, and preliminary assembly results that reflect the basic conditions of the sample genome are obtained. Then, compare the reading to the assembled genomic sequence and statistically map the distribution to the sequencing depth of the longest sequence, and finally, perform the comparative analysis of the obtained preliminary assembly results, screen the chromosomes and plasmid sequences, and their locations. Then, we used the GeneMarkS (Version 4.17) (Besemer et al., 2001) (http://topaz.gatech.edu/GeneMark/) to retrieve the related coding gene. The interspersed repetitive sequences were predicted using the RepeatMasker (Version open-4.0.5) (Saha et al., 2008) (http://www.repeatmasker.org/), and TRF (Tandem Repeats Finder, Version 4.07b) (Benson 1999) searched for tandem repeats in the DNA sequence. Transfer RNA (tRNA) genes were predicted by the tRNAscan-SE (Version 1.3.1) (Lowe and Eddy, 1997). Ribosome RNA (rRNA) genes were analyzed by the rRNAmmer (Version 1.2) (Lagesen et al., 2007). Small nuclearRNAs (snRNA) were predicted by BLAST against the Rfam (Version 1.1rc4) (Gardner et al., 2009; Nawrocki et al., 2009) database. The IslandPath-DIOMB (Version 0.2) (Hsiao et al., 2003) program was used to predict the Genomics Islands, and Prediction of prophages on the sample genome by phiSpy software (You et al., 2011) (Version 2.3) and the CRISPRdigger (Grissa et al., 2007) (Version 1.0) was used for the CRISPR identification.

We used seven databases to predict gene functions (genome annotated section, manuscript). A whole genome Blast search (E-value less than 1e-5, minimal alignment length percentage larger than 40%) was performed against above seven databases. The secretory proteins were predicted by the SignalP (Petersen et al., 2011) database, and the prediction of Type I-VII proteins secreted by the pathogenic bacteria were based on the EffectiveT3 (Eichinger et al., 2016) software. Meanwhile, we analyzed the secondary metabolism gene clusters by the antiSMASH (Medema et al., 2011). For pathogenic bacteria, we added the pathogenicity and drug resistance analyses. We used the PHI (Martin et al., 2015) (Pathogen Host Interactions), VFDB (Chen et al., 2012) (Virulence Factors of Pathogenic Bacteria), ARDB (Liu and Pop, 2009) (Antibiotic Resistance Genes Database) to perform the above analyses. Carbohydrate-Active enzymes were predicted by the Carbohydrate-Active EnZYmes Database (Cantarel et al., 2009).The SMRT portal (Version 2.3.0) (Konstantin et al., 2015; Sergey and Adam, 2015), the final genome assembly results were predicted by methylation site detection and possible methylation transferase recognition of nucleotide motifs and a circular gene distribution diagram obtained.

**References**

Benson G. Tandem repeats finder: a program to analyze DNA sequences[J]. Nucleic acids research, 1999, 27(2): 573.

Besemer J, Lomsadze A, Borodovsky M. GeneMarkS: a self-training method for prediction of gene starts in microbial genomes. Implications for finding sequence motifs in regulatory regions[J]. Nucleic Acids Research, 2001, 29(12): 2607-2618.

Cantarel B L, Coutinho P M, Rancurel C, et al. The Carbohydrate-Active EnZymes database (CAZy): an expert resource for glycogenomics[J]. Nucleic acids research, 2009, 37(suppl 1): D233-D238.

Chen L, Xiong Z, Sun L, et al. VFDB 2012 update: toward the genetic diversity and molecular evolution of bacterial virulence factors[J]. Nucleic acids research, 2012, 40(D1): D641-D645.

Eichinger V, Nussbaumer T, Platzer A, et al. Effective DB-updates and novel features for a better annotation of bacterial secreted proteins and Type III, IV, VI secretion systems. 2016, Nucleic Acids Res. doi:10.1093/nar/gkv1269.

Gardner P P, Daub J, Tate J G, et al. Rfam: updates to the RNA families database[J]. Nucleic acids research, 2009, 37(suppl 1): D136-D140.

Grissa I, Vergnaud G, Pourcel C. CRISPRFinder: a web tool to identify clustered regularly interspaced short palindromic repeats[J]. Nucleic acids research, 2007, 35(suppl 2): W52-W57.

Hsiao W, Wan I, Jones S J, et al. IslandPath: aiding detection of genomic islands in prokaryotes[J]. Bioinformatics, 2003, 19(3): 418-420.

Konstantin B, Sergey K, Chen-Shan Chin, et al. Assembling Large Genomes with Single-Molecule Sequencing and Locality Sensitive Hashing[J]. Nature Biotechnology 33, 623–630 (2015).

Lagesen K, Hallin P, Rødland E A, et al. RNAmmer: consistent and rapid annotation of ribosomal RNA genes[J]. Nucleic acids research, 2007, 35(9): 3100-3108.

Liu B, Pop M. ARDB-antibiotic resistance genes database[J]. Nucleic acids research, 2009, 37(suppl 1): D443-D447.

Lowe T M, Eddy S R. tRNAscan-SE: a program for improved detection of transfer RNA genes in genomic sequence[J]. Nucleic acids research, 1997, 25(5): 0955-964.

Martin U, Rashmi P, Arathi R et al. The Pathogen-Host Interactions database (PHI-base): additions and future developments. Nucleic Acids Research. 2015, doi: 10.1093/nar/gku1165.

Medema M H, Blin K, Cimermancic P, et al. antiSMASH: rapid identification, annotation and analysis of secondary metabolite biosynthesis gene clusters in bacterial and fungal genome sequences[J]. Nucleic acids research, 2011, 39(suppl 2): W339-W346.

Nawrocki EP, Kolbe DL, Eddy SR: Infernal 1.0: inference of RNA alignments. Bioinformatics 2009, 25(10):1335-1337.

Petersen T N, Brunak S, von Heijne G, et al. SignalP 4.0: discriminating signal peptides from transmembrane regions[J]. Nature methods. 2011, 8(10): 785-786.

Saha S, Bridges S, Magbanua Z V, et al. Empirical comparison of ab initio repeat finding programs[J]. Nucleic acids research, 2008, 36(7): 2284-2294.

Sergey K, Adam M P. One chromosome, one contig: complete microbial genomes from long-read sequencing and assembly[J]. Curr Opin Microbiol, 2015, 23:110-20.

You Z, YJ Liang, Karlene L, et al. “PHAST: A Fast Phage Search Tool” Nucl. Acids Res, 2011 doi:10.1093/nar/gkr485.

**Table S1** Effect of actinobacterial strain WZS021 on root physical parameters in two sugarcane varieties ROC22 and B8.

| Variety | Sampling time | Total length (m) | | Surf area (cm^2^) | | Volume (cm^3^) | | Root fresh weight (g) | | Root dry weight (g) | |
| --- | --- | --- | --- | --- | --- | --- | --- | --- | --- | --- | --- |
|  |  | WZS021 | Control | WZS021 | Control | WZS021 | Control | WZS021 | Control | WZS021 | Control |
| ROC22 | T1 | 2.03 ± 0.08** | 1.52 ± 0.04 | 43.87 ± 0.62*** | 35.52 ± 1.23 | 2.04 ± 0.34 | 1.72 ± 0.18 | 0.13 ± 0.02 | 0.14 ± 0.01 | 0.01 ± 0.00 | 0.01 ± 0.00 |
|  | T2 | 11.46 ± 1.11** | 5.77 ± 0.20 | 286.75 ± 12.66* | 248.08 ± 16.79 | 5.16 ± 0.53 | 4.61 ± 0.38 | 2.21 ± 0.33* | 1.18 ± 0.23 | 0.13 ± 0.01** | 0.07 ± 0.01 |
|  | T3 | 16.93 ± 0.77* | 13.51 ± 0.53 | 454.14 ± 27.98 | 420.64 ± 14.84 | 7.39 ± 0.44 | 6.40 ± 0.61 | 2.63 ± 0.64* | 2.23 ± 0.21 | 0.24 ± 0.01* | 0.20 ± 0.01 |
|  | T4 | 31.18 ± 1.28 | 29.12 ± 0.86 | 832.00 ± 20.96*** | 644.31 ± 24.41 | 12.66 ± 0.16** | 10.14 ± 0.28 | 9.16 ± 0.39*** | 5.40 ± 0.48 | 1.07 ± 0.04*** | 0.67 ± 0.02 |
| B8 | T1 | 2.20 ± 0.10** | 1.50 ± 0.04 | 29.52 ± 4.74 | 36.81 ± 3.22 | 1.71 ± 0.14* | 1.41 ± 0.09 | 0.15 ± 0.35* | 0.09 ± 0.11 | 0.02 ± 0.00** | 0.01 ± 0.00 |
|  | T2 | 8.24 ± 0.39** | 4.47 ± 0.56 | 225.66 ± 34.99 | 185.56 ± 11.74 | 3.10 ± 0.73 | 2.67 ± 0.48 | 1.23 ± 0.23** | 0.55 ± 0.08 | 0.09 ± 0.01** | 0.04 ± 0.00 |
|  | T3 | 15.53 ± 0.92* | 10.93 ± 0.61 | 355.65 ± 28.90* | 250.03 ± 32.15 | 5.02 ± 0.67 | 5.61 ± 0.22 | 2.41 ± 0.17*** | 1.00 ± 0.08 | 0.28 ± 0.02*** | 0.10 ± 0.01 |
|  | T4 | 27.13 ± 1.87* | 21.07 ± 0.93 | 666.22 ± 22.63*** | 375.27 ± 8.17 | 11.21 ± 1.20** | 7.47 ± 0.75 | 4.70 ± 0.30*** | 2.29 ± 0.24 | 0.52 ± 0.02*** | 0.29 ± 0.02 |
| Note: Experiment repeated three times and mean with standard deviation were used and significant (*p* < *0.05, **0.01, ***0.001) among treatments were calculated by *t-test*, T1-T4: 7, 14, 21, and 28 days after inoculation (DAI) | | | | | | | | | | | |

**Supplementary Figures**


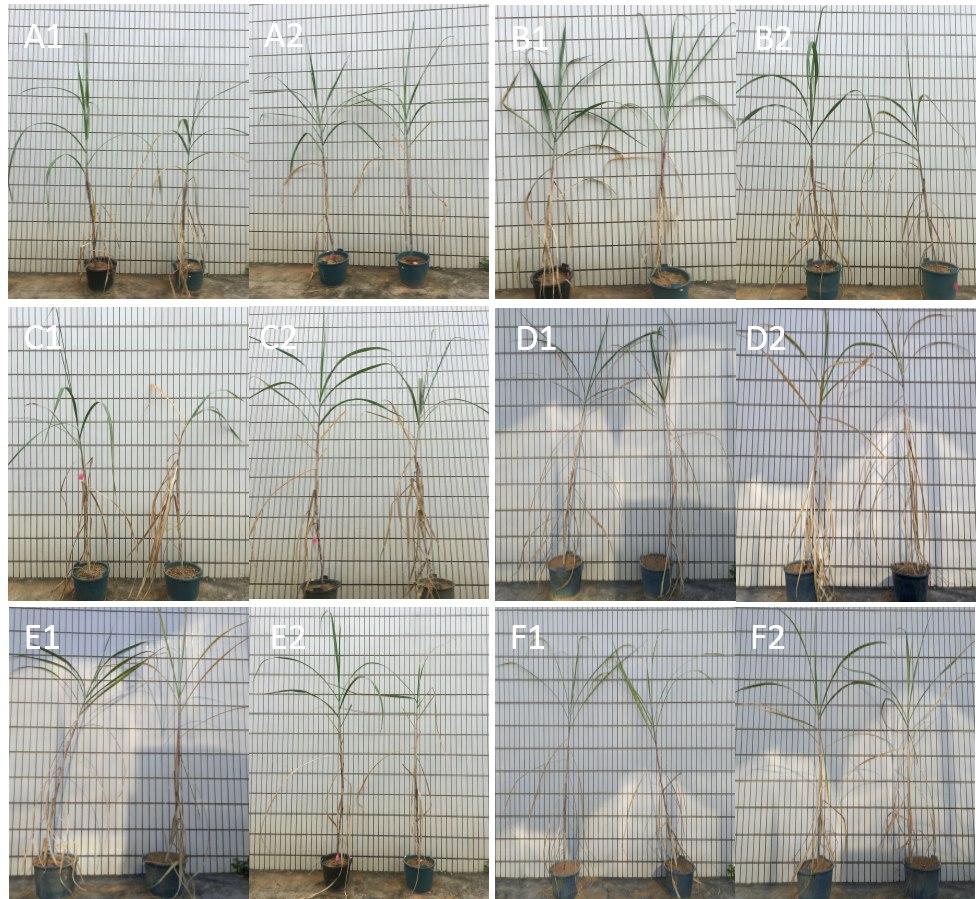


**Fig. S1** Comparison of external morphology between inoculated and non-inoculated plants. A-F: CK, D1, D2, D3, Rw1, Rw2; 1-2: ROC22, B8; left plant: inoculated; right plant: un-inoculated.


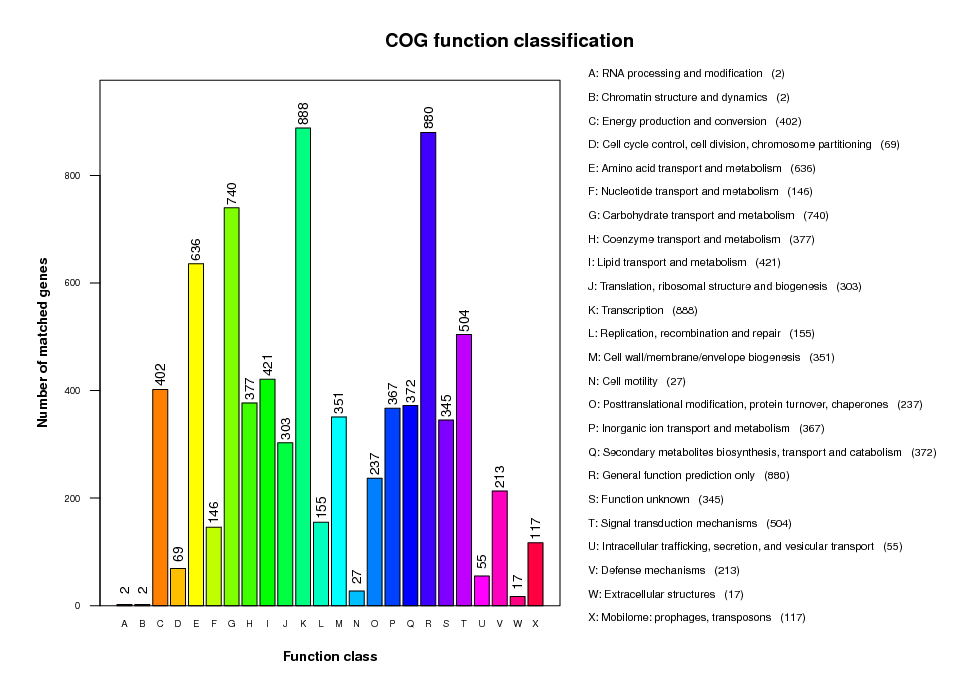


**Fig. S2** COG function classification of actinobacterial strain WZS021 genome


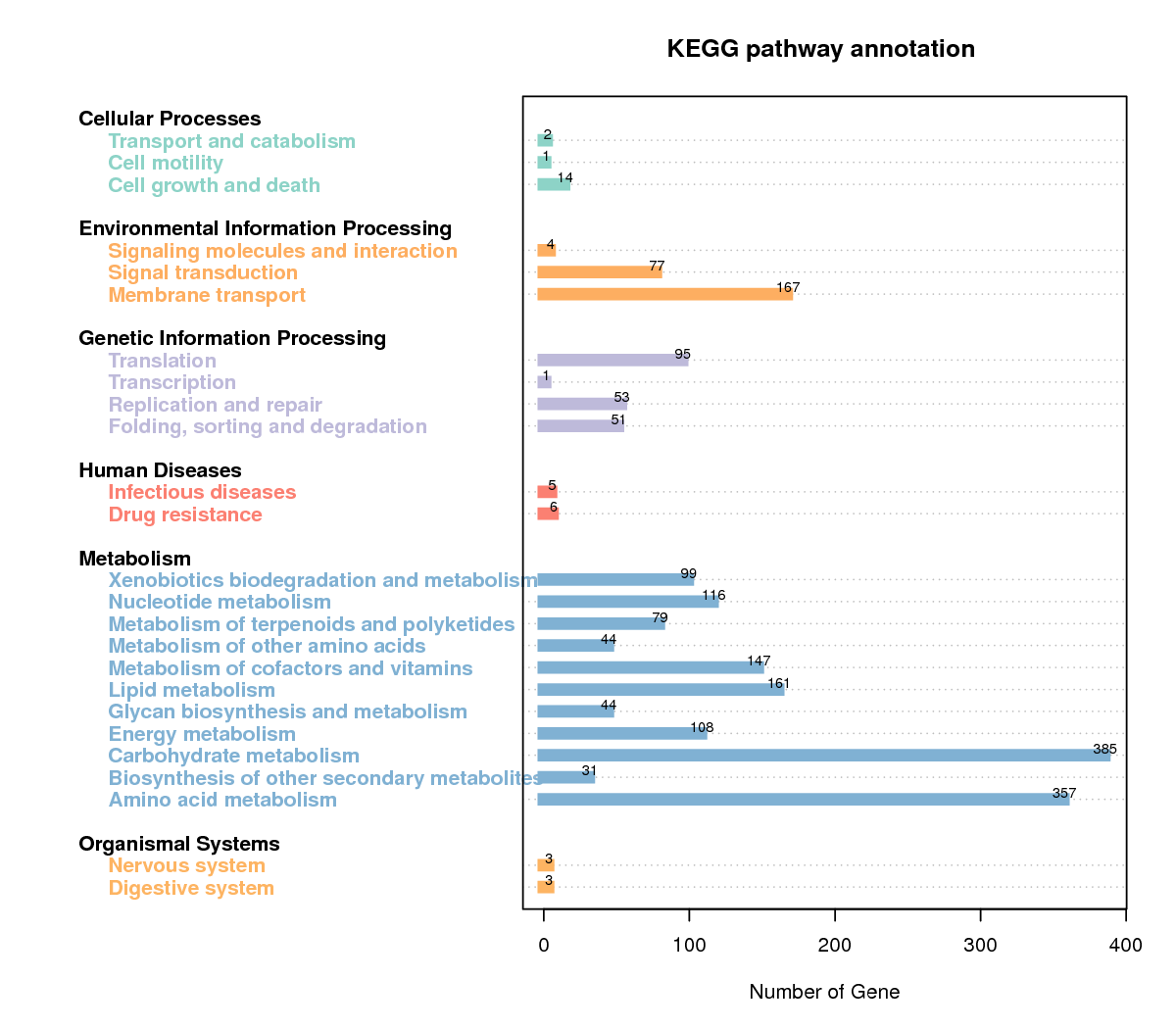


**Fig. S3** KEGG function classification of actinobacterial strain WZS021 genome


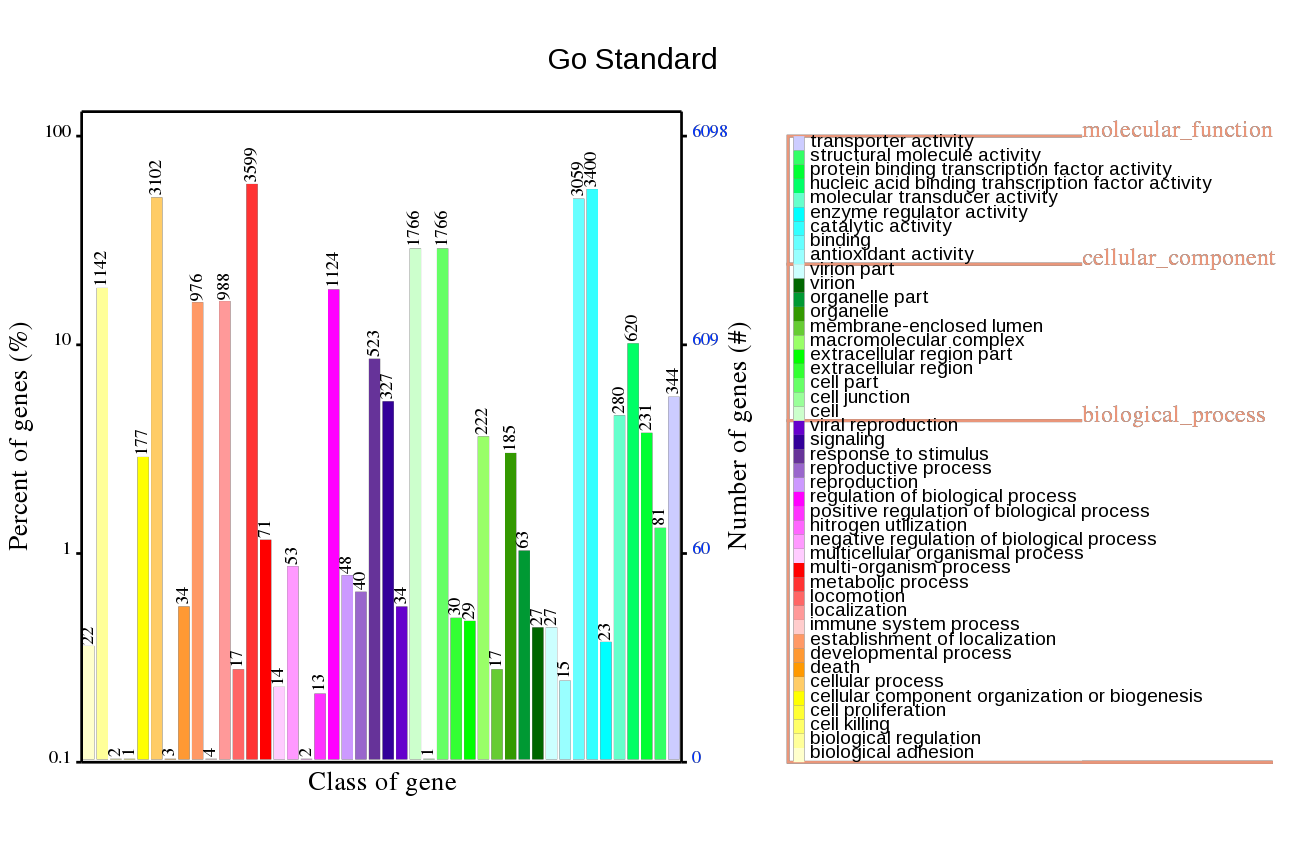


**Fig. S4** GO function classification of actinobacterial strain WZS021 genome
